# Special Issue—Spinal Cord Injuries: Advances in Rehabilitation

**DOI:** 10.3390/jcm13061782

**Published:** 2024-03-20

**Authors:** Mohit Arora, Ashley R. Craig

**Affiliations:** 1 Faculty of Medicine and Health, The University of Sydney, Sydney, NSW 2000, Australia; mohit.arora@sydney.edu.au; 2 John Walsh Centre for Rehabilitation Research, Kolling Institute, Northern Sydney Local Health District, St Leonards, NSW 2065, Australia

Spinal cord injury (SCI) is a severe, neurological disorder resulting from traumatic injury (such as a motor vehicle crash or fall) or non-traumatic injury associated with disease (such as cancer or infection) that results in impaired voluntary motor control and sensory function, usually leading to lifelong severe disability [1,2,3,4]. Secondary health conditions are common, compounding dysfunction and lowering quality of life [2,3], with prevalent conditions including autonomic nervous system dysfunction, cardiovascular disorder, cognitive impairment, bladder and bowel infection, skin disorders, sleep disorders, chronic pain and mental health disorder [2,3,4]. Consequently, people with SCI are vulnerable to experiencing barriers to achieving adjustment, such as inadequate resilience and coping skills to deal with the disability and any secondary health conditions [3,4,5,6]. Furthermore, social participation and social mobility can be impeded, family and social networks diminished and employment opportunities greatly lessened [4,6,7,8,9,10]. In such a complex and severe injury and coupled with the need for improved rehabilitation treatments and strategies, advances are required across the whole spectrum of SCI assessment and interventions that target the disability and secondary conditions.

SCI rehabilitation has been defined as a goal-oriented process constructed to optimize recovery of residual physical function, with the objective of gaining the highest possible level of personal adjustment, autonomy and independence [4,11]. To address the multiple challenges a person with SCI faces, it is crucial that the multidisciplinary health team (MDT) involved in SCI rehabilitation strategically collaborate to assist the person with SCI in attaining optimal recovery [4]. Additionally, the MDT must also work closely with social, educational and vocational services to achieve rehabilitation goals [11]. Evidence suggests that SCI rehabilitation resulted in improved management of secondary conditions as well as increased life expectancy in people with SCI [12]. However, the occurrence of disorders associated with SCI remains very high [4,5,6], and life expectancy is still well below that of the general population [13].

Given the above, it is critical that advances in SCI rehabilitation continue to occur to ensure optimal physical and psychological adjustment to SCI and that quality of life and life expectancy continue to improve. Arguably, one vital strategy for achieving this is through the appropriate communication of innovative research that features advances in SCI rehabilitation. With this in mind, researchers exploring strategies designed to promote innovation in SCI rehabilitation were invited throughout 2023 to submit their research to a Special Issue titled Advances in SCI Rehabilitation. We were extremely pleased with the response.

Thirteen high-quality papers written by highly experienced and internationally renowned researchers were accepted for publication in the Special Issue, covering the following topics:

(i) Innovative rehabilitation approaches to secondary conditions (Contribution 1: prediction of pressure ulcers after SCI; Contribution 2: investigation into sarcopenia after SCI the involuntary loss of skeletal muscle mass and strength and Contribution 3: a prospective study on the association between cognitive impairment after SCI and cognitive reserve before SCI).
(ii) Improvements in national approaches to screening and follow-up strategies (Contribution 4: evidence concerning national change in England in, among other things, screening for mental health, and Contribution 5: investigating changes to follow-up processes after SCI rehabilitation in the Netherlands).
(iii) Research into novel treatments that could provide essential advances in SCI rehabilitation (Contribution 6: a case study and laboratory framework on the possible beneficial effects of heart rate variability biofeedback and paced breathing after SCI; Contribution 7: evidence for the benefits of activity-based therapy for mobility and life quality after SCI; Contribution 8: research investigating restoration of walking after SCI using neuromodulation therapy; Contribution 9: a systematic review and meta-analysis on benefits of robot-assisted gait training after SCI; Contribution 10: a prospective study on the effects of robot-assisted gait therapy on mental health after SCI; Contribution 11: preliminary research on the beneficial effects of virtual reality (walking) for neuropathic pain after SCI; Contribution 12: a case study presenting evidence for beneficial effects of Hypnosis Enhanced Cognitive Therapy for pain after SCI and Contribution 13: a case study on the beneficial effects of spinal cord stimulation on autonomic dysreflexia after SCI.

These thirteen papers offer exciting SCI advances in rehabilitation. They all deal with a very complex disorder that results in debilitating life-long injury and impairment. These papers provide preliminary evidence that could deliver novel solutions to significant gaps in our understanding of the management of SCI. To highlight just a few.

Autonomic dysreflexia (AD) is a severe and potentially life-threatening syndrome after SCI involving abnormal reaction of the autonomic nervous system to sensory stimuli that provoke a sympathetic nervous system reflex. This then results in vasoconstriction that leads to dangerously increased blood pressure [14]. Treatments for AD are limited, and preliminary research with three individuals with SCI using epidural spinal cord stimulation at the level of the lumbosacral spinal cord showed great promise, reducing vascular sympathetic nervous system activation when provoked [14]. This type of therapy, therefore, has the potential to be used to reduce the risk of AD. Electrical stimulation was also used to restore standing and walking in individuals with SCI [15]. The case study results presented in the Special Issue [16] were based on an individual with chronic tetraplegia. This person received multiple weeks of stimulation training (e.g., standing, sitting up, treadmill walking and active cycling). The results were promising. The participant showed significant improvement in lower-limb volitional movements and, after the study, was to walk short distances with aids [16].

Chronic pain is a prevalent secondary condition after SCI, and treatments are limited and problematic, such as the overuse of opioid medications [17]. While pain management strategies like cognitive behavior therapies are known to be effective [18], these strategies are limited for individuals with SCI who are ventilator-dependent. Novel research that employed hypnotic cognitive therapy that relies less on verbal interaction showed promise for relieving pain symptoms in an individual with SCI [19].

The studies reported in this Special Issue are certainly cutting-edge and, importantly, informed by evidence-based groundbreaking research. They will, therefore, form the basis of future research that investigates critical gaps in our knowledge about SCI rehabilitation and play a pivotal foundation for transformative advances in SCI rehabilitation. The advances reported in this Special Issue will then hopefully be followed by a translation of findings into SCI rehabilitation programs around the world with the ultimate goal of enhancing the quality of life for people living with SCI worldwide.
